# Engaging With Hospital Staff to Develop Implementation Strategies For Delivering a Patient Falls Prevention Education Program Using a World Café

**DOI:** 10.1177/01939459261449623

**Published:** 2026-06-24

**Authors:** Cheng Yen Loo, Meg E. Morris, Jacqueline Francis-Coad, Steffanie Coulter, Hazel Heng, Ron Shorr, Uyen Phan, Carol Watson, Adam Semciw, Catherine M. Said, Bodil Rasmussen, Sarah Bunting, Christopher Etherton-Beer, Jeanette Wood, Anne-Marie Hill

**Affiliations:** 1 School of Health and Clinical Sciences, The University of Western Australia, Perth, WA, Australia; 2 WA Centre for Health & Ageing, The University of Western Australia, Perth, WA, Australia; 3 School of Allied Health, Human Services and Sport, La Trobe University, Bundoora, VIC, Australia; 4 Royal Perth Bentley Group, East Metropolitan Health Service, Perth, WA, Australia; 5 Allied Health, Northern Health, Melbourne, VIC, Australia; 6 Geriatric Research Education and Clinical Centre, North Florida/South Georgia Veterans Health System, Department of Epidemiology, University of Florida, Gainesville, FL, USA; 7 Physiotherapy Department, Western Health, St Albans, VIC, Australia; 8 Physiotherapy, Melbourne School of Health Sciences, The University of Melbourne, Parkville, VIC, Australia; 9 Australian Institute for Musculoskeletal Science (AIMSS), St Albans, VIC, Australia; 10 School of Nursing, Faculty of Health, Deakin University, Melbourne, VIC, Australia; 11 Centre for Quality and Patient Safety Research in the Institute for Health Transformation, Deakin University, Melbourne, VIC, Australia; 12 Nursing and Midwifery, Western Health, St Albans, VIC, Australia; 13 Medical School, The University of Western Australia, Perth, WA, Australia

**Keywords:** falls prevention, patient education, hospitals, care workforce, implementation strategy

## Abstract

**Background:**

Hospital falls can be reduced through patient and staff education, yet limited evidence exists about how staff can systematically implement patient falls prevention education. Planning implementation with staff may enhance their acceptance, engagement, and delivery of falls education to hospital patients.

**Objective:**

The objective of the study was to design an implementation plan with hospital staff to guide the successful delivery of patient falls education.

**Methods:**

Three participatory workshops using a world café methodology were conducted in 1 Western Australian and 2 Victorian hospitals. Participants were presented with information about a patient falls education program called “Safe Recovery” and discussed program implementation strategies. Conversation topics were staff education and training needs, ward support, and organizational requirements. Table discussions were captured on paper and analyzed iteratively at the forum. Subsequently, workshop field notes were analyzed using inductive content analysis.

**Results:**

Sixty-two hospital staff (n = 42 nurses, n = 12 allied health, n = 8 other) participated in the workshops. Participants considered the implementation process would be enabled at: (1) individual level, by providing accessible and flexible training to optimize staff engagement; (2) ward level, by establishing clear implementation protocols, engaging and supporting team leaders, and (3) ensuring clear communication between staff, patients, and families; and (4) organizational level, by leadership supporting sustained implementation. Group consensus was that it was important to have a single, agreed vision to implement the Safe Recovery Program.

**Conclusion:**

Staff engagement facilitated the development of a shared vision and structured plan to implement a patient falls prevention education program on hospital wards.

## Introduction

Falls are among the most common hospital adverse events^1^ with 25% to 50% causing injury and contributing to longer stays, higher costs, and increased discharge of older patients to residential care.^2-6^ Australian hospitals spend an estimated $590 million AUD each year on fall-prevention efforts,^7^ and hospitals in the United Kingdom invest around £1.1 billion.^8^ Despite this substantial spending, falls continue to occur frequently, particularly among older patients.

World and Australian falls guidelines strongly recommend the use of patient and staff education and implementing multifactorial strategies based on a personalized risk assessment.^9,10^ These recommendations concur with research trials that have found patient education with supportive staff education is effective for reducing hospital falls and injuries.^11-15^ However, a global review found major gaps in implementing evidence-based falls guidelines, including limited attention to patient education and little guidance on how to deliver it in practice.^16^ This gap in the translation of evidenced-based practice in the area of patient falls education strongly suggests that more research that examines implementation in this area is required.

Implementation frameworks are recognized as providing healthcare systems with a comprehensive suite of plans that address important aspects of translating evidence into practice.^17^ These frameworks embed the theory that successful implementation requires strong and systematic stakeholder engagement, that is, engaging stakeholders in all stages of a project. Systematic engagement of staff throughout the development, delivery, and evaluation of the planned implementation can facilitate the identification of context-specific barriers to implementation and propose acceptable and feasible strategies to promote translation.^18^

An evidence-based falls prevention education program titled the Safe Recovery Program (SRP) has been shown to reduce falls and injuries and was positively received by older patients in hospital.^19^ The program uses adult learning and behavior change theory to inform patients about risk of falls and strategies to reduce their risk of falls while in hospital. The program is delivered and reinforced by ward staff to allow staff and patients to work together to prevent falls.^20,21^ The SRP was recently revised and updated through consumer and hospital staff engagement and co-design.^22^ The revised program was found to be acceptable to consumers and staff, and increased older patients’ knowledge and motivation to engage in fall-prevention strategies.^22^

In recent studies, older consumers and staff were engaged to explore barriers and enablers to implementation of the revised SRP on hospital wards.^5,23,24^ Both stakeholder groups concurred that raising patients’ awareness of falls risk, and staff personalizing delivery of education for each patient and delivery by dedicated staff were key facilitators to effective implementation.^5,23,24^ Staff additionally identified the need for falls champions, staff education about falls prevention, and hospital systemic support.^5,23^ Meanwhile, older consumers emphasized the importance of using high-quality resources, including those in other languages, timely delivery during admission, and having clear communication to build confidence.^24^ Program preparation for delivery subsequently commenced with the appointment of 2 ward champions (1 clinical nurse and 1 physiotherapist) to train allied health assistants who would deliver the initial education materials to patients, and support ward staff to assist patients to engage and respond to the education.^5,23,24^ We previously conducted a novel RCT that found using allied health assistants was a feasible approach to initiate program delivery.^15^ Allied health assistants (AHAs) provide support services based on the clinical reasoning of allied health professionals, and work under allied health professional supervision to deliver care that augments service capacity, accessibility, and efficiency.^25^

However, there was limited evidence for how to effectively implement the strategies developed by staff and consumers. While the SRP has been previously evaluated for its effectiveness for reducing falls in randomized trials^11,12,26^ and qualitative research has identified barriers and enablers to program delivery,^5,19,23,27^ no studies have yet developed an implementation plan for translating the SRP into a real-world clinical setting. The Consolidated Framework of Implementation Research (CFIR) is one theory of implementation.^28^ This theory provides a framework that can guide systematic assessment of potential barriers and facilitators to implementing new health programs. Assessment of factors affecting implementation can then be used to design a tailored implementation plan.^17,28^ Using this framework, we aimed to develop a clear implementation plan would enable translation of the recommended strategies in a systematic manner across the participating hospital wards. The objective of the study was to design an implementation plan between the researchers and hospital health professionals on participating wards to guide the successful delivery of the SRP.

## Methods

### Design

Three hospital staff workshops were conducted using the world café methodology.^29,30^ This qualitative method facilitates participant engagement in constructive dialog around critical questions^31^ enabling the cross-pollination of ideas in an equitable and non-threatening environment. Unlike a focus group or individual interview where staff are passive participants and do not contribute to data analysis, this approach facilitated timely active collaboration between hospital staff to engage in problem identification, and used collective intelligence to contribute to the planning and design of pragmatic solutions to support the effective implementation of patient falls prevention education within hospital wards.^30^

### Participants and Setting

Adopting a purposive sampling strategy, hospital staff were eligible to participate if (a) they worked on or managed surgical or medical wards that were eligible to implement the SRP and (b) had sufficient English language skills to engage in conversation. Hospital management from the relevant sites advertised the workshops in face-to-face meetings and emails. Hospital management then provided a list of prospective participants for the research team to formally invite via email to attend the staff workshop. Reminder emails were sent 3 weeks prior to each workshop to encourage participation from staff who had not yet confirmed their availability. From all 3 hospitals, a total of 104 staff members were invited to participate, of whom 62 accepted. One workshop was held in Western Australia, and 2 in Victoria. The workshops were held at each of the 3 sites to allow local ward staff to collaborate, thereby fostering future team collaboration upon program commencement.

### Discussion Topics

The discussion topics explored staff requirements for hospital implementation of the SRP and to devise solutions to facilitate program delivery on hospital wards. A structured discussion guide with additional probing questions was created for the workshop hosts (Supplemental Appendix 1). Topics were initially scoped by the researchers on the team (A.-M.H., M.E.M., and J.F.-C.) who had extensive, expert knowledge on falls prevention education and developed in discussion with clinical researchers on the team (C.W., A.S., C.M.S., S.C., and H.H.) who worked in the participating hospitals in falls prevention roles, such as quality and safety falls committees.

The 4 topics discussed were designed to address the implementation process domain of the CFIR framework^17^ with focus on engaging with staff (as frontline deliverers of the education program) to identify strategies that would enable delivery. We took a multi-level organizational approach to encourage the group to identify strategies at an individual staff, ward, and organizational level:

1. How to effectively deliver falls prevention training and education to staff and patients at their hospital?
2. What ward-level support is needed to enable effective delivery of the education program at each hospital site?
3. What communication processes need to be developed at each hospital site?
4. What organizational support is needed at each hospital site?

### Workshop Procedure and Data Collection

The workshop was conducted according to the 7 core principles of the world café: (1) setting the context; (2) creating a hospitable space; (3) exploring relevant questions; (4) encouraging everyone’s contribution; (5) connecting diverse perspectives; (6) listening for patterns and insights; and (7) sharing collective discoveries.^29,31^

The lead facilitators in Western Australia (A.-M.H.) and Victoria (M.E.M.) were senior researchers, recognized experts on hospital falls prevention, and had extensive prior clinical experience.^15,32,33^ The research team had strong participatory methods experience, including in world café methodology.^22,34,35^ Workshop planning included liaising with the participating hospitals through members of the research team (C.W., S.C., H.H., A.S., U.P., C.M.S., B.R., and S.B.), who were clinical researchers and clinical falls experts, leading engagement. The lead facilitators provided the table hosts, who were expert clinicians in the area with research experience, with training on the world café method, the falls education program to be discussed, and procedures for conducting the workshop.

The same procedure was used for each of the 3 workshops. The workshops were hosted in a private conference room set up to create an inviting café-style atmosphere. A member of the research team welcomed participants and invited them to sit at a table in groups of 6 to 7. All participants completed a consent form and demographic survey and were offered light refreshments throughout the event.

Each workshop ran for approximately 3 hours and was managed by a timekeeper. Each workshop commenced with the lead site facilitators providing a 20-minute overview of the research project, the SRP, and aims of the workshop. Each discussion round lasted 20 minutes, and every table discussed all 4 topics. The group discussion and feedback was audio-recorded for data triangulation during the analysis.

Table hosts moderated discussions, prompting participants to brainstorm and record ideas on sticky notes, while notetakers transcribed responses and hosts captured collective ideas on butcher paper. This procedure followed the suggested methodology for research world cafés with the aim to add research rigor.^30^ At the end of each discussion topic, all written materials were collected for analysis (J.F.-C., M.E.M., C.M.S., H.H., and A.-M.H.), and the tables commenced with new butcher paper. After each round, the lead facilitators instructed participants to switch tables to encourage the cross-pollination of ideas. At the end of the workshop, approximately 20 minutes were allocated to a whole group discussion, where the master analysts presented a summary of the preliminary findings for participants to review, confirm, and validate. Each topic summary was presented to the group verbally by 2 analysts and visually on screen, with each table invited to comment to the whole group, while the third analyst made notes. An edited version was created in real time and further verbal, summative comments from participants invited and added alongside. These final versions for each topic were displayed to the café participants who were invited to add any final feedback or clarification.

### Data Analysis

All written materials were collated and transcribed verbatim into Microsoft Excel spreadsheets. Audio recordings were transcribed verbatim using an online transcription service and checked for accuracy by the researchers. Inductive content analysis, a qualitative research method of making valid inferences from data in relation to their context, was used to interpret and quantify patterns, themes, and meanings within textual content.^36,37^ This enabled the research team to identify the presence, frequency, and relationship of specific elements in data, as little was known about hospital staff’s thoughts on implementing the SRP on medical and surgical wards.

Five research assistants supervised by 3 experienced researchers (A.-M.H., J.F.-C., and C.Y.L.) reviewed data spreadsheets independently multiple times to familiarize themselves with the data. Data from all workshops were merged for each topic prior to category creation and abstraction. Responses were transferred onto coding sheets to form multiple categories, which were then streamlined by grouping and merging, inspired by the CFIR.^17,23,28^ Input from other research team members was sought in assigning content-specific terms to each category. Related subcategories were combined into broader generic categories, which led to the identification of a main category that reflected group consensus.

### Rigor and Trustworthiness

Credibility was demonstrated through member checking of response accuracy and interpretation by workshop participants at the close of discussion rounds and participation of 3 independent researchers in data analysis. Dependability was determined through the provision of an audit trail, and confirmability was established through verbatim responses representing participant voices.^36,38^ The study design and conduct are reported using the Consolidated Criteria for Reporting Qualitative Studies (COREQ) guidelines.^39^

### Ethical Considerations

Ethics approval for the study was obtained from the Royal Perth Hospital Human Research Ethics Committees (ID: RGS 7007), Northern Health Human Research Ethics Committee (ID: 2025_Non-HREC_08), and Western Health Low Risk Ethics Panel (ID: LR/25/WH/116003). All participants provided written informed consent.

## Results

Participants’ demographic characteristics are presented in Table 1. A total of 62 health professionals from Western Australia (one world café, n = 21) and Victoria (2 world cafés, n = 41) participated in the 3 workshops. Staff recruited were comprised of clinical ward staff, staff educators, and hospital managers. The largest professional group represented was nurses (n = 42; 67.7%), followed by physiotherapists (n = 12; 19.4%). Most participants worked mixed (day/night) shifts (n = 39; 62.9%) and were involved in direct patient care (n = 37; 59.7%).

**Table 1.** Workshop Participant Demographics.

Staff demographics	Workshop A, n = 21	Workshop B, n = 22	Workshop C, n = 19	Total, n = 62
**Gender, n (%)**				
Male	2 (9.5%)	2 (9.1%)	2 (10.5%)	6 (9.7%)
Female	19 (90.5%)	20 (90.9%)	17 (89.5%)	56 (90.3%)
**Age range, n (%)**				
20-34	9 (42.9%)	6 (27.3%)	7 (36.8%)	22 (35.5%)
35-44	6 (28.6%)	8 (36.4%)	5 (26.3%)	19 (30.7%)
45-54	4 (19.1%)	4 (18.2%)	7 (36.8%)	15 (24.2%)
55-64	2 (9.5%)	4 (18.2%)	0 (0.0%)	6 (9.7%)
**Profession, n (%)**				
Physiotherapist	7 (33.3%)	2 (9.1%)	3 (15.8%)	12 (19.4%)
Nurse	14 (66.7%)	16 (72.7%)	12 (63.2%)	42 (67.7%)
Occupational therapist	0 (0.0%)	2 (9.1%)	3 (15.8%)	5 (8.1%)
Allied health assistant	0 (0.0%)	1 (4.5%)	0 (0.0%)	1 (1.6%)
Physician	0 (0.0%)	1 (4.5%)	1 (5.3%)	2 (3.2%)
**Education level, n (%)**				
Bachelor’s degree	13 (61.9%)	13 (59.1%)	6 (31.6%)	32 (51.6%)
Graduate certificate or diploma	2 (9.5%)	8 (36.4%)	2 (10.5%)	12 (19.4%)
Post-graduate qualification	6 (28.6%)	1 (4.5%)	11 (57.9%)	18 (29.0%)
**Language spoken at home, n (%)**				
English	16 (76.2%)	18 (81.8%)	16 (84.2%)	50 (80.7%)
Other	5 (23.8%)	4 (18.2%)	3 (15.8%)	12 (19.4%)
**Employment status, n (%)**				
Full time	17 (81.0%)	4 (18.2%)	11 (57.9%)	32 (51.6%)
Part time	4 (19.1%)	18 (81.8%)	8 (42.1%)	30 (48.4%)
**Shifts normally worked, n (%)**				
Mixed (day/night) shifts	14 (66.7%)	15 (68.2%)	10 (52.6%)	39 (62.9%)
9 am-5 pm (not shift)	7 (33.3%)	7 (31.8%)	9 (47.4%)	23 (37.1%)
**Role, n (%)**				
Direct patient care	13 (61.9%)	16 (72.7%)	8 (42.1%)	37 (59.7%)
Management on ward	5 (23.8%)	3 (13.6%)	9 (47.4%)	17 (27.4%)
Administration/executive	3 (14.3%)	3 (13.6%)	1 (5.3%)	7 (11.3%)
No response	0 (0.0%)	0 (0.0%)	1 (5.3%)	1 (1.6%)
**Work specialty, n (%)**				
Medical wards	11 (52.4%)	7 (31.8%)	8 (42.1%)	26 (41.94%)
Surgical/Orthopedic	4 (19.1%)	0 (0.0%)	2 (10.5%)	6 (9.68%)
Other^a^	6 (28.6%)	15 (68.2%)	9 (47.4%)	30 (48.39%)
**Time in profession, n (%)**				
0-2 years	5 (23.8%)	5 (22.7%)	0 (0.0%)	10 (16.1%)
3-5 years	1 (4.8%)	4 (18.2%)	3 (15.8%)	8 (12.9%)
6-10 years	6 (28.6%)	2 (9.1%)	5 (26.3%)	13 (21.0%)
>11 years	9 (42.9%)	11 (50.0%)	10 (52.6%)	30 (48.4%)
No response	0 (0.0%)	0 (0.0%)	1 (5.3%)	1 (1.6%)
**Falls education in previous 12 months, n (%)**				
Yes	6 (28.6%)	11 (50.0%)	9 (47.4%)	26 (41.9%)
No	15 (71.4%)	11 (50.0%)	9 (47.4%)	35 (56.5%)
No response	0 (0.0%)	0 (0.0%)	1 (5.3%)	1 (1.6%)

a Other includes gerontology, rehabilitation, mental health, oncology, and critical care.

**Figure 1.**
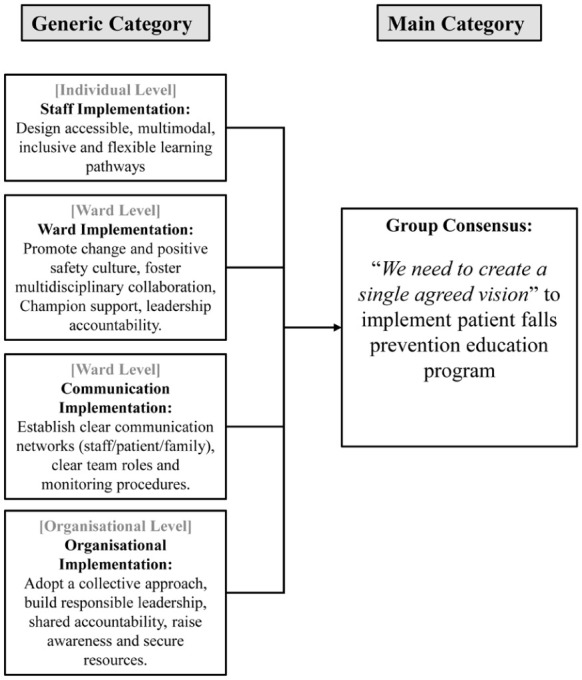
Pathway to group consensus.

Staff feedback is presented by workshop (workshops named as A, B, and C) and cross-referenced by the table number within each workshop (Tables 1–3). Four generic categories, comprising the implementation strategies, emerged from the discissions and the group consensus identified the main category as “a single agreed vision was required to deliver the program.” Categorization is summarized in Figure 1.

The implementation strategies addressing the individual (staff), ward, and organizational levels are presented below and overall, formed the implementation plan.

### Category 1: Provide Flexible and Inclusive Training to All Hospital Staff (Individual Level)

There was agreement that hospital staff on the wards that would be delivering the program should receive training about the SRP because “it’s important to help get everyone [staff] on the same page and working together” (A3). Many participants suggested training should include all ward staff, not just allied health and nurses, such as students, clerical staff, physicians, and cleaners. According to A2, “cleaners are in and out of rooms and notice things. . . it’s important they do some training and understand the importance of decluttering corridors.” Many participants noted that the nature of hospital shift work made it essential to provide training in a “flexible way to accommodate new, rotating staff and those who work weekends” (A1; A3; B1). To serve as a reminder to staff of their role in supporting program delivery, B3, and C3 suggested “adding SRP information as the screensaver on ward computers” and including “updates on the hospital intranet iNews” (C2).

#### 1a: Offer Accessible Education Training Options

Acknowledging the need for accessible, multimodal training, many participants said offering diverse learning options for staff was important (C1, C2, A2). Taking an opportunistic approach to deliver training, some participants said, “some wards hold daily face-to-face meetings, which could incorporate training into the agenda” (A3, C1). Other participants suggested, “delivering training during the nurse or safety huddles, at ward, staff, and multidisciplinary team meetings” (A3, C2, C3). Providing communication updates during the bed-based transition care program meetings was also identified (B2).

However, delivering staff training solely in person was not considered practical. According to participants in A3, “the challenge is knowing when staff are available because different people tend the wards every week” (A3). Some participants suggested “providing training information on the ward journey board where daily updates and patient progress reports are listed” (A1). Training at a distance was also suggested by “developing e-training modules that could be stored in a central location, such as the ward’s Toolbox learning package” (A2). Uploaded training into the “learning management system mandatory training platform and in-service” (C2, C3) was also suggested.

#### 1b: Deliver Education at an Appropriate Duration, Pace, and Frequency

Participants discussed how training could be delivered to maximize staff engagement and learning. Some participants had concerns about “finding time to do the training” (A1). Meanwhile, several participants said it was “important to reinforce the training by having frequent repeats to keep falls prevention a priority” (A2, C2).

Overall, there was consensus that staff training should be delivered using different strategies to suit diverse learning styles. One group suggested introducing a “central repository where all staff across different hospital sites could access digital training” (B3). On a local level, “integrating falls prevention training into their existing hospital e-learning platform” was deemed appropriate (B2).

#### 1c: Motivate Staff Engagement in Training

Exploring solutions to motivate staff, several participants said, “Unless the training was mandatory. . .I probably wouldn’t do it” (A2; B3). Experiencing e-learning fatigue was also mentioned, “we already have a lot of online training . . .we don’t like it” (A2). To maintain staff motivation, it was suggested “short training modules ranging from 10 to 20 minutes would make it easier for staff” (A2, C2). Meanwhile, A2 said, “I always go for the short ones first [training modules]. . .it keeps things engaging.” This was reinforced by other participants who said, “You don’t want to attend a presentation where 50 minutes later you’re tired and sleepy” (C1. A3).

### Category 2: Engage and Support Teams (Ward Level)

#### 2a: Foster a Positive Workplace Culture at a Ward and Hospital Level

Workshop participants said fostering a positive workplace culture was key to creating a culture of falls prevention in hospital wards. As the AHA cohort would be delivering the SRP education to patients, participants said, “we need to make the AHAs feel part of the team” and recognize them as a “valuable part of the workplace delivering the SRP program” (A2). Echoing this sentiment, offering positive reinforcement by saying “well done team” (A1) and “inspiring staff with positive role modelling” were recommended (C2, B2).

#### 2b: Engage Support From the SRP Champions

The SRP champions were seen as a link to encourage collaboration among the multi-ward staff and teams caring for patients. According to B3, “it’s important we have someone we can reach out to.” Their visibility was considered important to provide ongoing staff training about the SRP, motivate staff to deliver the education to patients, and be a sounding board to “trouble-shoot barriers, answer difficult questions, and help with complex patients” (A2, B1). High visibility and presence of the SRP champions were deemed important during program commencement to “make sure the falls prevention strategies that the program promotes are in place” (A3) and be a “sounding board for staff” (A1, C2).

Workshop group A2 and C1 said the SRP champions should “regularly meet with the team (hospital staff) to teach and advertise the program.” Regarding the staff training, it was important for the “Safe Recovery champions to be available when rolling out the training” (A2). Exploring how to make the SRP champions’ presence known, it was suggested “they should be made visible with distinctive uniforms and easily contactable” (A3). Querying further, about being easily contactable, it was suggested that the SRP champions should be “reachable on pagers or by using their MS Teams chat” (A2; A3, B2, C1, C3).

#### 2c: Engagement of Ward-Level Leaders

Garnering support from ward-leaders to help maintain the momentum of delivering education to patients was raised by workshop participants. One group suggested “ward-leaders should set achievable goals” and “liaise closely with the champions”(B2). Showing ongoing interest by “monitoring program participation and following up with staff” (B3) was also mentioned.

### Category 3: Ensuring Clear Communication Between Staff, Patients, and Family (Ward Level)

Participants discussed clarifying staff roles and improving communication with staff, patients, and families.

#### 3a: Affirming the Patient Plan

Exploring how staff could encourage the enactment of patient plans on the ward, several strategies were proposed to assist staff, including “providing updates on the journey board regularly” (A1), “updating the electronic clinical handover record regularly” (A1), and “embedding reminders to check the patient plans into the electronic medical record” (A2, C1).

Other ideas included photocopying the patient SRP plan so that “one set could be placed at the patient’s bedside and another on the observation chart” (A2). Laminating the patient plan template so they could be updated regularly (A3, B2, C1) was also considered, along with investing in a “wipeable board that could be updated” (A2). All participants emphasized having the patient plan “easily accessible and clearly displayed in the patient’s room” as a priority (C1, C2). While staff agreed on multiple ways to support patients, they acknowledged that no single approach fits all wards.

#### 3b: Communication With Patients and Family

Effective communication with patients and their families was explored among workshop participants. Many participants said, “staff need to talk with the patients and listen” (A2, B1). This included making patients feel welcome to ask questions about the SRP. Many participants said, “having the SRP video was good for people who aren’t able to read” (B3). When delivering the education to patients from non-English speaking backgrounds, “interpreters and the translated SRP resources should be used” (A2, C3). Furthermore, many participants said “AHAs should be made to feel confident finding and using interpreters” (B1). Some participants suggested “increasing the pool of in-person interpreters” (B2) to better represent the larger cultural groups admitted to hospital.

To assist family and carers become aware of the SRP, “posters should be made visible on the wards and include a QR code for them to access the information” (B2, B3). There was also consensus that “the patient plan should be discussed when family is present” (C1, C2) and ensure they too are “responsible for educating their family member [the patient]” (C3). It was suggested that “conversations with the patient and family should start from pre-admission or when they arrive at emergency” (C1, C3). To help patients feel settled, “family should be encouraged to stay” (C1) because “they should be part of education sessions” (C2). In instances where family/carers are not present, group B2 suggested “getting consent from the patient to phone to their next of kin.”

Providing information about the SRP and involving family/carers was deemed important because “family can reiterate the plan with the patient and translate information in ways that the patient understands” (C1). This included drawing on family to “sell the messaging to the patient” (C1) and “translate information to the language spoken by the patient” (C3). During pre-admission, it was also suggested that the family could be involved by “asking them to bring appropriate footwear/gait-ware and clothing” for the patient (C1).

#### 3c: Define Ward-Level Roles and Responsibilities

There was consensus that clarity around roles and responsibilities needed to be established. This included knowing “which patient on the ward has received the education by documenting this information in the EMR and journey board” (B2). As the plan was for the SRP to be delivered by AHAs to patients, there was consensus that the AHAs should receive training and orientation for each hospital ward where the program would be delivered. As part of the ward orientation, “AHAs need to know who the important staff are in each ward and for staff to be aware of the AHAs” (A3). To reinforce roles and responsibilities, participants at tables A1, A2, and B2 suggested including AHAs in nurse safety huddles, ensuring awareness of the role of nursing support, and holding daily face-to-face check-ins.

Participants also said that it was important to have an “established supervision system for AHAs with clear policies, protocols, and procedures” (A1, C1). This included being clear about which ward meetings AHAs should attend, establishing clear handover procedures, and knowing when AHA should co-sign documents such as the patient care plan.

#### 3d: Establish Procedures For Effective Communication and Performance Monitoring

While suggestions varied slightly between hospital sites, there was agreement that staff required clear and concise instructions for effective communication about the SRP between staff. Some participants said, “communication should occur during handovers, weekly multidisciplinary team meetings, nurse huddles, staff and ward meetings” for regular updates (C1, C2). “The journey board is a good place to communicate information (about patient plans) with large groups of staff” (A3). However, there was also a strong preference for verbal communication between staff and the strategic placement of posters as reminders for staff about the SRP.

Participants on A3 and C2 said daily handovers were required “to assist in the identification of new and current patients to receive the education.” In group C1, some participants said “adding prompts on the structured communication framework” would be helpful, along with “building in reminders on the electronic medical record” (C1). To support this process, one group said, “most wards have admission and discharge books, which could be used to identify new patients to receive the education” (A3).

Communication between staff to ensure shared understanding was discussed. Some participants suggested “adding a note on the patient file, creating a list of patients yet to be seen or placing a note at the patient’s bedside table where it’s visible” (A1, A2). Documenting the “patient’s plan on the EMR and having a tick-box option to mark education delivery” was also suggested (C1, C2). However, all workshops concurred that local adaptations would be required.

### Category 4: Leveraging Multi-Level Hospital Support to Achieve Success (Organizational Level)

Many participants identified the need for organizational support in the form of tangible resources and responsible leadership to ensure implementation success.

#### 4a: Tangible Resources

To support the successful implementation of the SRP, there was a strong emphasis on gaining allyship from hospital leadership to support the program. This included suggesting environmental improvements such as having “different models of call bells for patients” unable to use the standard bells (A1), investing in “more high-back chairs” to assist patient ambulation from a sitting position (A1), and “increasing the number of tablets and headsets” on each ward for patient viewing of the SRP video (A2, C1, B2). Access to the “ward televisions to play the SRP video on repeat” (A2, B3) and assistance from IT to “enable far-reaching access to training resources on the global email and training hub” (A3) were deemed important.

#### 4b: Organizational Ownership

To guide the successful delivery of the SRP, there was agreement that support was needed at the hospital organizational level, where “everyone takes ownership” of the program by “creating buy-in bottom-up, middle-out, and top-down” (A1, C1). Some participants said making the SRP a standing agenda item in meetings backed by nurse unit managers and heads of department would help encourage staff to “be on board” with the program (A1, B2). Reinforcing this sentiment, another table said, “We need to have senior management to remind staff about the importance of this program” (A2, B2). Securing endorsement at the highest level of hospital management included an “email from the CEO, and having the program featured in divisional meetings” to help create hospital-wide participation (C3).

Senior leaders were considered important to “help create a shared reality” (A1). Echoing this sentiment, another group said, “it’s all about closing the loop and helping motivate staff” (B1). This included communicating the SRP throughout the hospital institution and “presenting information at the comprehensive care committee and directors” (A2). Other participants also said senior leaders should “be involved in ensuring that it is all implemented in the most optimized manner.”

### Main Category: A Single Agreed Vision Required For Implementation

The main category emerged from group consensus. Overall, the combined consensus was that there was a need to create a single agreed vision to deliver the SRP to patients. Workshop A stated, “there should be ownership by all,” while Workshop B suggested “everyone should participate, because it’s everybody’s job.” In Workshop C, participants stressed the need for staff to “understand the program and its purpose, set clear expectations and plan for long-term sustainability.”

Participants agreed that a collective, team-based approach supported by shared accountability, flexible multimodal training, clear communication networks, and strong leadership would enhance program delivery and fidelity. Fostering a positive ward culture that celebrates success would help motivate staff to be more engaged.

## Discussion

Engaging hospital health professionals in discussion using a world café methodology enabled the design of an implementation plan to guide the successful delivery of patient falls education in hospitals. Health professionals identified pragmatic strategies that were analyzed and synthesized into an overall plan.^23,28^ Participants, as key stakeholders, were able to provide local, informed suggestions for strategies, making these strategies more likely to be feasible to implement, and thereby increasing the adoption of the planned education program.^18,40^ Overall, the participants said successful implementation would require delivering flexible training for staff using champions, along with having ward and organizational support. Participants stressed the importance of establishing clear communication channels on an individual, ward, and organizational level. This multi-level approach reflects evidence that effective implementation depends on the program, its settings, the people involved, and strong processes for planning, engagement, execution, and evaluation, consistent with the CFIR. Strategies provided by the group, such as using champions, informing local leaders, involving patients, and making training dynamic, align with expert recommendations for implementing change, suggesting they would facilitate implementation.^40^

Across all workshops, participants stated that providing staff with training was highly sought after, which underscores the absence of falls prevention training being systematically delivered to staff in hospitals.^16,41^ Previous studies have found that delivering patient education effectively requires training to be provided to hospital staff.^13,32^ Providing staff training is designed to ensure clarity on how to effectively deliver education to patients while gaining endorsement and a shared sense of ownership of program implementation. A recent Delphi study^42^ recommended that staff and patient education be delivered regularly, with appropriate resources and dedicated time allocated to support this education.

Many participants said staff training should be diverse, accessible, and offer multi-modal learning to accommodate shift work and include repeated engagement. This aligns with concepts of adult learning, where learning should apply to the learner’s work and other responsibilities valued by the learner.^43^ Findings were supported by our earlier research that identified that a single one-hour in person or online session was not sufficient to develop confidence to provide patient falls education.^13^ Several studies have found that incorporating diverse learning pathways for nursing students was positively linked to critical thinking skills.^44,45^ Based upon workshop feedback, an overwhelming majority said offering an in-person education component was very important, particularly during the early stages of program implementation.

Streamlined communication and clear ward-level roles were seen as essential for successful implementation. Fast-paced hospital environments and high staff turnover can weaken ownership of patient education.^5,23,46^ Evidence shows clinicians often view nurses as primarily responsible for falls prevention, highlighting the need for stronger inter-professional communication and organizational support to reinforce that patient education is a shared responsibility.^47^

To affirm delivering falls prevention education as a responsibility of all hospital staff, the participants agreed it was important to foster a workplace culture that advocated for a positive safety culture.^21^ Ensuring high visibility of SRP champions on the wards was perceived as an effective strategy to keep falls prevention education a key priority for staff, while helping to troubleshoot problems and overcome ward-level barriers.

Recommendations to ensure patients’ families could easily access the plans align with previous studies emphasizing family involvement in patient education.^5,23,27^ The ward-level recommendations highlighted the importance of considering workforce redesign to ensure the timely and practical delivery of the SRP in hospital wards.^14,15^ Organizational-level recommendations of requiring hospital leaders to take ownership also accord with the need to recognize hospital as complex healthcare settings.^43^

### Strengths and Limitations

A strength was the involvement of diverse hospital staff who were to be directly involved in the education program implementation on their wards; hence, their insights enhanced the credibility of SRP implementation decisions. Involving staff stakeholders improves the likelihood of eventual adoptability and feasibility. Implementation science frameworks explain that stakeholder engagement improves feasibility by ensuring real-world grounding of the strategies and identifying practical challenges prior to commencement.^48^ Using a world café to engage staff can be useful for planning implementation as it provides an opportunity for group co-creation of strategies in a rigorous and timely manner and builds ongoing ownership of the implementation.^17,30,35^

The study has several limitations. A key limitation is that the implementation plan was created but has not been evaluated. Further research that evaluates the implementation of the SRP and concurrently evaluates the effectiveness of wards using the strategies identified in this study is underway. A world café has some limitations, including that follow-up group feedback is not feasible to obtain, meaning that the implementation plan is finalized by the research team. Table hosts’ observations and leadership can potentially introduce bias, and we aimed to minimize possible bias by the researchers providing training for table hosts prior to the event.^30^ Dominant participants at tables can potentially sway the discussion and to mitigate this limitation we followed the procedure of ensuring rotation of participants between rounds. The implementation plan was limited to English-speaking participants in Australia and on wards at 3 hospitals, highlighting the need for future studies across other hospitals, broader settings and cultures. While context-specific, our findings may be useful for other hospitals seeking to implement patient falls education programs. The strategies provide detailed information for clinical staff about strategies that could be evaluated in other settings and potential barriers to address, including the need for a shared vision and ownership of falls prevention by the whole team.

## Conclusion

Hospital staff and researchers designed an implementation plan to enable the delivery of patient education on hospital wards to reduce patient falls and fall-related injuries. Engaging hospital staff to prepare a structured implementation plan prior to commencement of a patient falls education program can enhance the delivery of falls prevention education and contribute to its successful implementation.

## Supplemental Material

sj-pdf-1-wjn-10.1177_01939459261449623 – Supplemental material for Engaging With Hospital Staff to Develop Implementation Strategies For Delivering a Patient Falls Prevention Education Program Using a World Café Supplemental material, sj-pdf-1-wjn-10.1177_01939459261449623 for Engaging With Hospital Staff to Develop Implementation Strategies For Delivering a Patient Falls Prevention Education Program Using a World Café by Cheng Yen Loo, Meg E. Morris, Jacqueline Francis-Coad, Steffanie Coulter, Hazel Heng, Ron Shorr, Uyen Phan, Carol Watson, Adam Semciw, Catherine M. Said, Bodil Rasmussen, Sarah Bunting, Christopher Etherton-Beer, Jeanette Wood and Anne-Marie Hill in Western Journal of Nursing Research
