# Supplementary material for: Engaging With Hospital Staff to Develop Implementation Strategies For Delivering a Patient Falls Prevention Education Program Using a World Café
Source: West J Nurs Res. 2026 Jun 24;48(8):865–75. doi: 10.1177/01939459261449623 (PMC13365306; doi:10.1177/01939459261449623)
Supplement: sj-pdf-1-wjn-10.1177_01939459261449623 – Supplemental material for Engaging With Hospital Staff to Develop Implementation Strategies For Delivering a Patient Falls Prevention Education Program Using a World Café [file sj-pdf-1-wjn-10.1177_01939459261449623.pdf]

## Appendix 1: Staff workshop discussion guide

<table>
<tr><td>Topic 1</td><td>Education and Training, virtual Community of Practice (vCoP), Online Platform and Safe Recovery Program (SRP) champions<br>Note: the vCoP is a virtual meeting place online for champions from multiple hospitals and contains resources and training programs<br>Probing questions:<br>• Who do you think should engage in the vCoP with the falls champions?<br>• How regularly should vCoP online meetings convene?<br>• What format should the training be for the ward staff?<br>  o How long should the training be for Allied Health/Nurses/Medical?<br>  o When is the best time to provide training for Allied Health /Nurses/Medical?<br>  o What medium should the training be delivered to Allied Health /Nurses/Medical?<br>• What type of training resources would you like to have access to on the vCoP?<br>• What do you think the online training should include?</td></tr>
<tr><td>Topic 2</td><td>Delivery of Safe Recovery Program on Wards<br>Probing questions:<br>• How can Allied Health Assistants be supported to do the first delivery of the program? Timing?<br>• Where will patients write/leave their plan?<br>• How will Allied Health /Nursing communicate the plan? To each other/ to patients?<br>• How will Allied Health supervise Allied Health Assistants?<br>• How will patients be supported/communicated with by all staff to write and enact their plan?<br>  o What added support/communication needs are required to cater to Culturally and Linguistically Diverse (CALD) population?<br>  o What resources required?<br>  o How could family/ carers be utilised?</td></tr>
<tr><td>Topic 3</td><td>Ward support and communication for program<br>Probing questions:<br>• What ward administrative / communication processes are needed to effectively deliver the Safe Recovery Program<br>• What is the best way to disseminate information about the SRP to Allied Health staff and Nurses in your ward?<br>• What resources could help make it easier to deliver the SRP in your hospital ward?<br>• What type of support do you think Allied Health and Nursing Staff would need from the Safe Recovery Program Champions in your ward?<br>• For patients who are cognitively impaired– who will check if they should receive the program / improve and can receive program?</td></tr>
<tr><td>Topic 4</td><td>Organisational Support<br>Probing questions:<br>• What barriers exist in your hospital ward that might make it difficult to deliver the Safe Recovery Program to patients and their families?<br>• What type of infrastructural support and facilities could help make it easier to deliver the Safe Recovery Program?<br>• How and what level of involvement do you think senior managers need to be involved to advocate and support implementation of the SRP?</td></tr>
</table>
